# QLT0267, a small molecule inhibitor targeting integrin-linked kinase (ILK), and docetaxel can combine to produce synergistic interactions linked to enhanced cytotoxicity, reductions in P-AKT levels, altered F-actin architecture and improved treatment outcomes in an orthotopic breast cancer model

**DOI:** 10.1186/bcr2252

**Published:** 2009-05-01

**Authors:** Jessica Kalra, Corinna Warburton, Karen Fang, Lincoln Edwards, Tim Daynard, Dawn Waterhouse, Wieslawa Dragowska, Brent W Sutherland, Shoukat Dedhar, Karen Gelmon, Marcel Bally

**Affiliations:** 1 Advanced Therapeutics, BC Cancer Agency, 675 West 10th Avenue Vancouver, British Columbia, V5Z 1L3, Canada; 2 Department of Pathology and Laboratory Medicine, University of British Columbia, 2329 West Mall, Vancouver, British Columbia, V6T 1Z4, Canada; 3 QLT, Inc., 887 Great Northern Way, Vancouver, British Columbia, V5T 4T5, Canada; 4 Faculty of Pharmaceutical Sciences, University of British Columbia, 2329 West Mall, Vancouver, British Columbia, V6T 1Z4, Canada; 5 Cancer Genetics, BC Cancer Agency, 675 West 10th Avenue Vancouver, British Columbia, V5Z 1L3, Canada; 6 Department of Biochemistry, University of British Columbia, 2329 West Mall, Vancouver, British Columbia, V6T 1Z4, Canada; 7 Medical Oncology, BC Cancer Agency, 675 West 10th Avenue Vancouver, British Columbia, V5Z 1L3, Canada

## Abstract

**Introduction:**

Substantial preclinical evidence has indicated that inhibition of integrin linked-kinase (ILK) correlates with cytotoxic/cytostatic cellular effects, delayed tumor growth in animal models of cancer, and inhibition of angiogenesis. Widely anticipated to represent a very promising therapeutic target in several cancer indications, it is increasingly evident that optimal therapeutic benefits obtained using ILK targeting strategies will only be achieved in combination settings. The purpose of this study was to investigate the therapeutic potential of the ILK small molecule inhibitor, QLT0267 (267), alone or in combination with chemotherapies commonly used to treat breast cancer patients.

**Methods:**

A single end-point metabolic assay was used as an initial screen for 267 interactions with selected chemotherapeutic agents. These *in vitro *assays were completed with seven breast cancer cell lines including several which over-expressed human epidermal growth factor receptor 2 (Her2). One agent, docetaxel (Dt), consistently produced synergistic interactions when combined with 267. Dt/267 interactions were further characterized by measuring therapeutic endpoints linked to phosphorylated protein kinase B (P-AKT) suppression, inhibition of vascular endothelial growth factor (VEGF) secretion and changes in cytoarchitecture. *In vivo *efficacy studies were completed in mice bearing orthotopic xenografts where tumor growth was assessed by bioluminescence and calliper methods.

**Results:**

The combination of 267 and Dt resulted in increased cytotoxic activity, as determined using an assay of metabolic activity. Combinations of cisplatin, doxorubicin, vinorelbine, paclitaxel, and trastuzumab produced antagonistic interactions. Further endpoint analysis in cell lines with low Her2 levels revealed that the 267/Dt combinations resulted in: a three-fold decrease in concentration (dose) of 267 required to achieve 50% inhibition of P-AKT; and a dramatic disruption of normal filamentous-actin cellular architecture. In contrast to Her2-positive cell lines, three-fold higher concentrations of 267 were required to achieve 50% inhibition of P-AKT when the drug was used in combination with Dt. *In vivo *studies focusing on low Her2-expressing breast cancer cells (LCC6) implanted orthotopically demonstrated that treatment with 267/Dt engendered improved therapeutic effects when compared with mice treated with either agent alone.

**Conclusions:**

The findings indicate that the 267/Dt drug combination confers increased (synergistic) therapeutic efficacy towards human breast cancer cells that express low levels of Her2.

## Introduction

Integrin-linked kinase (ILK), an intracellular serine/threonine kinase, is a key signaling molecule expressed in most, if not all, tissues, with high levels of expression in normal pancreatic, cardiac and skeletal muscle tissues. Through interactions with a diverse range of proteins including adapters such as particularly interesting Cys-His-rich protein (PINCH), calponin homology-containing ILK-binding protein (CH-ILKBP), affixin and paxillin, kinases such as integrin-linked kinase-associated serine/threonine phosphatase 2C (ILKAP), protein kinase B (AKT) and phosphoinositide-dependent kinase 1 (PDK-1), and transmembrane receptors such as β1 and β3 integrins [1], ILK is thought to play a key role in integrin and growth factor receptor related signaling cascades [2,3]. For example, ILK acts as a scaffold protein to allow for protein-complex formations connecting extracellular integrin signals to intracellular actin cytoskeleton rearrangements through direct interaction with the cytoplasmic domain of β1 integrin [4]. Cell extracellular matrix (ECM) adhesion complexes influence a vast number of cellular processes including cellular morphology, migration, proliferation, survival, and differentiation. Activation of downstream targets of ILK such as AKT [5], glycogen synthase kinase 3 (GSK-3) [6], myosin light chain (MLC) [7], affixin [8] and the cytoplasmic domain of β1 integrin [9], is associated with signaling cascades known to regulate transcription of genes involved in a diverse range of functions including: cell survival, cell cycle progression, cell adhesion and spreading, focal adhesion plaque formation, ECM modification, cell motility, and contractility [1,10].

Increased ILK expression and activity is found in association with many cancer types including: breast, brain, prostate, pancreatic, colon, gastric, ovarian, and malignant melanomas [4,11-16]. Further, there is mounting experimental evidence indicating that ILK plays a pivotal role in many processes associated with tumorigenesis. Enforced over-expression of ILK in immortalized rat intestinal epithelial cells induces epithelial to mesenchymal transition (EMT) and a transformed tumorigenic phenotype that is, in part, linked to ILK-dependent inhibition of E-cadherin expression and increased nuclear translocation of β catenin. Over-expression and constitutive activation of ILK leads to dysregulated growth and suppression of apoptosis and anoikis [17,18]. With specific respect to breast cancer, over-expression of ILK in mammary cells stimulates anchorage-independent cell growth, cell cycle progression, and increased cyclin D and A expression *in vitro *[2,19]. Furthermore, mammary epithelial cells over-expressing ILK exhibit hyperplasia and tumor formation *in vivo*. [4]. Further evidence has indicated ILK might play a key role in VEGF-mediated endothelial activation and angiogenesis [4,20].

Targeted inhibition of ILK in cancer cells by various strategies can also lead to suppression of the AKT signaling pathway, inhibition of cell cycle progression, reduced vascular endothelial growth factor (VEGF) secretion *in vitro*, and reduced tumor growth *in vivo *[21]. A number of pharmaceutically viable small-molecule inhibitors of ILK have been developed and partially characterized. From the K15792 class of the pharmacophor family [22], some of these inhibitors were shown to effectively inhibit cancer cell survival, growth [23] and invasion [24], and induce apoptosis and cell-cycle arrest *in vitro *[25], as well as inhibit tumor growth and angiogenesis *in vivo *[20]. Interestingly, the most promising ILK inhibitor, QLT0267 (267), while capable of eliciting pleiotropic effects in xenograft models of glioma, was unfortunately shown to only delay, but not prevent, tumor growth *in vivo*, even at doses as high as 200 mg/kg [2,23]. Based on these findings, we speculate that optimal therapeutic effects of 267 will only be realized using a combination therapeutic strategy.

Here we demonstrate on the basis of a cell viability assessment determined using multiple breast cancer cell lines that 267 in combination docetaxel (Dt) interacted in a synergistic manner (increased therapeutic benefit over single agents as assessed by the median effect methodology developed by Chou and Talalay [26]). Experimentations aimed to identify underlying molecular mechanisms and additional drug-drug interactions using multiple endpoint analyses, revealed in breast cancer cells expressing low levels of Her2, beneficial drug-drug interactions on the basis of endpoints measuring AKT phosphorylation and F-actin cytoarchitecture. Using an orthotopic model of breast cancer (low Her2), 267/Dt combinations were found to exert enhanced therapeutic activity, as demonstrated by significantly reduced tumor growth and extended survival in mice treated with the combination compared to the single agents.

## Materials and methods

### Chemicals

Cisplatin, doxorubicin, paclitaxil, Dt, vinorelbine, and trastuzumab (Tz) were obtained from the British Columbia Cancer Agency Pharmacy (Vancouver, BC, Canada) and 267 was a generous gift from QLT Inc (Vancouver, BC, Canada). All other chemicals, unless specified, were purchased from Sigma Chemical Company (Oakville, Ontario, Canada). Dt was reconstituted in 13% ethanol for a final concentration of 10 mg/ml and Tz (Hoffman-La Roche, Mississauga, Ontario, Canada) was reconstituted in PBS at a stock concentration of 21 mg/ml.

### Cell-lines and culture

MCF-7, KPL-4, BT-474, MDA MB/468 and SKBR3 cells were purchased from American Type Culture Collection (Manassas, VA, USA). MDA-MB-435 (LCC6) estrogen receptor-negative breast cancer cells [27] and MCF-7^Her2 ^cells were generously donated. LCC6^Her2 ^cells, previously described by our group [28], were generated by the stable transfection (neomycin selection using G418) of plasmid DNA containing the *Her2 *gene driven by the cytomegalovirus promoter. LCC6 cells were stably transfected using a lenti-virus system with the luciferase gene and green fluorescent protein (GFP). Cells were sorted by FLOW cytometry for GFP expression and selected cells were used in the following experiments. Sorted cells exhibited similar *in vitro *and *in vivo *growth rates as the parental LCC6 cell line. Additionally LCC6^luc ^and parental LCC6 were equally sensitive to Dt.

The breast cancer origin of the LCC6 parental cell line, MDA-MB-435, is controversial. Based on studies of Ross and colleagues [29] and Rae and colleagues [30] it has been suggested that the MDA-MB-435 cell line is of a melanoma origin. However, Sellappan and colleagues [31] have been able to demonstrate that MDA-MB-435 cells can be induced to express breast differentiation-specific proteins and secrete milk lipids. Further, more recent studies of Neve and colleagues [32] have demonstrated that the MDA-MB-435 cell line shares many molecular features with breast cancer cell lines of breast epithelium origin. In studies from our laboratory [28] using a LCC6 cell line permanently transfected with the *Her2 *gene (LCC6^Her2 ^cell line), we have been able to demonstrate that the Her2-positive variant exhibit enhanced survival under stress, overproduction of VEGF, activation of nuclear factor (NF) κB and *in vivo *sensitivity to Tz (aka Herceptin™; Hoffman-La Roche, Mississauga, Ontario, Canada); results that are consistent with what is known about Her2-positive breast cancer models. Thus, we believe it is justifiable to use these cells as a model breast cancer cell line; particularly when the results obtained using this cell line are confirmed with other breast cancer cell lines.

LCC6, LCC6^Her2^, LCC6^luc^, KPL-4, BT-474, MDA MB/468, MCF-7 and MCF-7^Her2 ^cells were maintained in Dulbecco's modified eagle's medium (DMEM)/high glucose supplemented with L-glutamine (2 mmol/L; DMEM and L-glutamine from Stem Cell Technologies, Vancouver, BC, Canada) 5 mM penicillin/streptomycin, and 10% FBS (Hyclone, Logan, UT, USA). SKBR3 cells were maintained in McCoy's 5a medium (fStem Cell Technologies, Vancouver, BC, Canada) supplemented with L-glutamine, 5 mM penicillin/streptomycin, and 10% FBS. All cells were maintained at 37°C and 5% carbon dioxide in a humidified atmosphere.

### Cell viability assays

Metabolic activity (measure of cell viability) of breast cancer cell lines incubated in the presence of various therapeutic agents was determined using Alamar Blue^® ^assays (Medicorp Inc. Montreal, QU, Canada) according to the manufacturer's suggestions. Briefly, 6000 cells/well seeded in triplicate onto 96-well flat-bottom tissue culture plates (Techno Plastic Products AG, Trasadingen, Switzerland) were allowed to adhere to the substratum for 24 hours under normal growth conditions (37°C and 5% carbon dioxide in a humidified atmosphere). Serial dilutions of individual drugs, 267/drug combinations and vehicle controls diluted in appropriate cell culture medium were then added to the wells and cells were grown for an additional 72 hours. To assess cell viability, cells were then incubated with 10% resazurin solution for four hours at 37°C and fluorescence was measured at 560/590 nm using an Optima fluorescence plate reader (BMG Labtech, Durham, NC, USA). Relative fluorescence determined from drug-treated cells was normalized to fluorescence determined from control cells (cells grown in presence of appropriate vehicle control alone) and data is shown as percentage relative cell viability compared with vehicle-treated control cells (100% viability, highest fluorescence). Background fluorescence was subtracted from all samples and results of experiments conducted in triplicate are indicated (average ± standard deviation).

### Drug combination effects – median effect principle

To determine whether various 267/drug combinations had resulted in synergistic, antagonist, or additive effects, the median effect principle (MEP) method of Chou and Talalay was used to determine combination index (CI) values [26,33,34]. Briefly, the MEP method is used to describe and understand the relationship between a measured response within a population of cells (fraction affected (f_a_) versus the fraction unaffected (f_u_)) and the fraction of the dose (D) required to achieve an effect level of 50% and is represented by the formula:



- where *D*_m _is the dose required to achieve a 50% effect level and m is a coefficient indicating the sigmoidicity of the dose-effect curve. The right side of the equation [(D/D_m_)^m^] represents the dose, and the left side of the equation [*f*_a_/*f*_u_] represents the effect of the interaction. The CI can be calculated at any effect level and the effect used can be derived on the basis of different endpoints (e.g. cell viability, inhibition of VEGF secretion, etc.). If CI is equal to one then the combination interactions result in additive effects, if the CI is less than one the combination interactions are considered synergistic, and if the CI is greater than one the combination interactions are considered antagonistic.

To determine CI values, the commercially available program CalcuSyn (Biosoft Ferguson, MO, USA) was used to calculate CI values for a broad range of effect levels and, on the basis of this analysis, F_a _versus CI plots were generated. CI values were then used to estimate the dose reduction index (DRI) for combination of drugs. The DRI estimates the extent to which the dose of one or more agents in the combination can be reduced to achieve effect levels that are comparable with those achieved with single agents. Drug combinations that acted synergistically can be identified as those that exhibited significant dose reduction values (i.e. a given measured effect will be observed at dose(s) significantly lower than expected based on single agent activities).

### VEGF expression

To determine whether a specified treatment influenced VEGF expression, ELISA assays using Quantikine Human VEGF Immunoassay kits (R&D Systems, Minneapolis, MN, USA) were conducted according to manufacturer's suggestions. Briefly, 6000 cells were seeded onto 96-well tissue culture plates and allowed to adhere for 24 hours. Cells were then grown in the presence of single agents or combinations of drugs for 72 hours (as described above). The experiments were completed in triplicate and repeated at least two times. Supernatants were collected, combined, and then assayed for the presence of secreted VEGF (specific for recombinant human VEGF_165 _and recombinant human VEGF_121_) using the Optima fluorescence plate reader (BMG Labtech, Durham NC, USA). Results were normalized to total protein found in supernatant and compared with standard curves determined using VEGF standards provided in the kit. This assay accurately measures VEGF levels between 9 pg/ml and 2000 pg/ml.

### Western blot analysis

Total protein lysates were prepared from cells incubated in the presence of single drug, the drug combinations or vehicle controls. Briefly, cells were rinsed with PBS, harvested from plates with trypsin, and centrifuged at 1500 × g for five minutes. Cell pellets were then re-suspended in lysis buffer (150 mmol/L sodium chloride, 1% NP40, 0.5% sodium deoxycholate, 2.5 mmol/L EDTA, 0.1% SDS), Mini protease inhibitor cocktail tablets (Roche Diagnostics, Mannheim, Germany), sheared using 25-gauge needles, incubated on ice for 30 minutes, and finally centrifuged at 10,000 × g for 10 minutes to remove insoluble material. Protein concentrations were determined from supernatant using the Bradford Method and approximately 75 μg of total protein from each sample were denatured in loading buffer (Invitrogen, Burlington, ON, Canada) by boiling for 10 minutes and loaded onto 10% SDS-PAGE. Proteins separated by electrophoresis were transferred to Nitrocellulose membrane (Millipore, Bedford, MA, USA) and blocked for one hour at room temperature in Odyssey blocking buffer (Licor Biosciences, Lincoln, NB, USA). Membranes were incubated at 4°C overnight in Odyssey blocking buffer containing polyclonal anti-ILK, anti-AKT, anti-P-AKT or anti-Her2 antibodies (1:1000 dilution; Cell Signalling Technology, Beverly, MA, USA). Membranes were then washed three times for five minutes with PBS-Tween (1% v/v) and incubated with either anti-rabbit or mouse IRDYE (green) (Rockland, Gilbertsville, PA, USA) or anti-rabbit Alexa 680 (red) (Invitrogen, Molecular probes, Burlington, ON, Canada) (1:10,000) for one hour at room temperature and signals were detected and quantified using the Odyssey Infrared Detection System and associated software (Odyssey v1.2; Licor, Lincoln, NB, USA). Background and input variation between samples were corrected using signal intensities for negative control pixel noise and actin band intensities, respectively. Data were expressed as mean values ± standard deviation and parametric analysis was performed using an unpaired Student t-test.

### Immunofluorescence analysis

Cells grown on coverslips were rinsed with PBS (pH 7.4), fixed using 2.5% paraformaldehyde (w/v) in PBS for 20 minutes at room temperature and permeabilized using 0.5%Triton X-100 (v/v) in PBS for five minutes at room temperature. Coverslips were then washed three times with PBS and incubated for one hour in 2% BSA (w/v) in PBS to block non-specific binding, washed three times in PBS, and then incubated with phalloidin conjugated to Texas red (1:500) (Molecular Probes, Eugene, OR, USA) for 20 minutes at room temperature. Nuclei were stained using Hoechst nuclear stain (10 mg/ml) (Molecular Probes, Eugene, OR, USA) (1:1000) for 15 minutes at room temperature. Coverslips were rinsed once with double distilled water and mounted to microscope slides using a 9:1 solution of glycerol and PBS (Air Products & Chemicals, Inc., Allentown, PA, USA). Images were viewed and captured using a Leica CTR-mic UV fluorescent microscope (Wetzlar, Germany) and a DC100 digital camera with Open Lab software (Improvision, Lexington, MA, USA).

### Tumor xenografts

All animal studies were conducted in accordance with institutional (University of British Columbia) guidelines for humane animal treatment and according to the current guidelines of the Canadian Council of Animal Care. Mice were maintained at 22°C in a 12-hour light and dark cycle with *ad libitum *access to water and food. Two million LCC6^luc ^cells were injected into the mammary fat pad of female NCr nude mice (Taconic, Oxnard, CA, USA) in a volume of 50 μL using a 28-gauge needle. Tumor growth was monitored using an IVIS 200 non-invasive imaging system (Xenogen, Caliper Life Sciences, MA, USA), and manually using callipers when tumor dimensions exceeded 3 mm in length and width. Tumor volume (mm^3^) estimated from length and width measurements were calculated according to the equation length times width squared divided by two with the length (mm) being the longer axis of the tumor. Animal body weights were recorded every Monday and Friday.

### *In vivo *imaging system

Imaging was performed once every seven days to monitor tumor progression. LCC6^luc ^tumor-bearing mice were injected intraperitoneally with 500 μl D-luciferin (15 mg/ml) (Xenogen Corp., Alameda, CA, USA). Mice were anesthetized using isoflurane and twenty minutes post intraperitoneally injection mice were imaged. Photographic and luminescence images were taken at exposure times of one, two, and five second(s) and Xenogen IVIS^® ^software was used to quantify non-saturated bioluminescence in regions of interest (ROI). Light emission between 5.3067 × 10^6 ^and 2.2179 × 10^9 ^was determined to contain tumor tissue while emissions below this range were considered as background. Bioluminescence was quantified as photons/second/cm^2^/steradian for each ROI.

### Statistical analysis

All statistical data was collected using GraphPad InStat (San Diego, CA, USA). One-way analysis of variance was performed using standard error of the mean, mean and n and a Tukey-Kramer Multiple Comparisons Test was used as the *post hoc *test.

## Results

### Breast cancer cells treated with 267 exhibit dose-dependent decreases in cell viability

**Figure 1:**
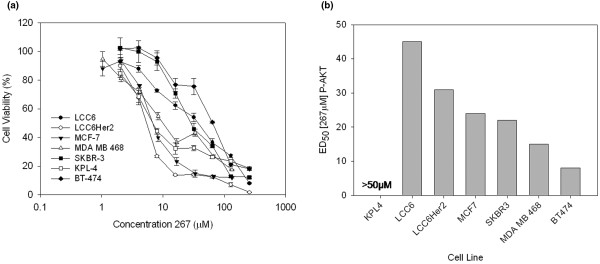
Breast cancer cells exhibit dose-dependent decrease in (a) cell viability and (b) P-AKT in response to increasing concentrations of the ILK small molecule inhibitor, QLT0267. **(a) **Seven breast cancer cell lines (LCC6, LCC6^Her2^, SKBR-3, KPL-4, BT-474, MBA-MB-468, and MCF-7) were treated with increasing doses (1 μm to 256 μM) of 267 for 72 hours and cell viability was evaluated using the AlamarBlue^® ^assay. Percentage cell viability relative to control (untreated) cells are shown. Each data point represents the mean (± standard deviation) determined from three experiments done in triplicate. Treated cells were assessed for phosphorylated protein kinase B (P-AKT) using western blot analysis of protein lysates collected eight hours after treatment. **(b) **Western blot were analyzed using densitometry, dose-response curves were generated and analyzed using Calcusyn to determine the ED50 of 267 for PAKT suppression for each cell line. 267 = QLT0267; ILK = integrin-linked kinase.

To study whether inhibition of ILK causes reduced breast cancer cell viability, seven human breast cancer cell lines (BT474, SKBR-3, KPL-4, MDA-MB/468, MCF-7, LCC6 and LCC6^Her2^) were exposed to serial dilutions (ranging from 1 to 256 μM) of the small molecule inhibitor of ILK, 267. As shown in Figure 1a, all cell lines examined exhibited 267 dose-dependent decreases in cell viability. Using the CalcuSyn™ program, effective doses (ED) capable of eliciting a 10, 50, or 90% decrease in cell viability were extrapolated from each dose response curve and these data have been summarized in Table 1. ED values showed some variation (ED_50_s ranging from 10 to 71 μM) depending on the specific breast cancer line examined. In general, slower growing breast cancer cells (SKBR-3 and BT-474) appear less sensitive to 267 than faster growing breast cancer cells (MCF-7 and LCC6).

**Table 1:** Effective doses at 10, 50 and 90% effect levels of QLT0267 in human breast tumour cell lines

**Cell line**	**Drug concentration range (μM)**	**ED10 (μM)**	**ED50 (μM)**	**ED90 (μM)**
MDA MB 435/LCC6	2.0 to 256.0	2.05	17.2	143.6
MDA MB 435/LCC6her2/*neu*	2.0 to 256.0	1.66	12.2	90.4
MDA MB 468	1.0 to 133.3	0.91	25.6	718.7
MCF-7	1.0 to 133.3	1.15	9.8	83.3
KPL-4	2.0 to 256.0	0.31	12.1	473.0
BT-474	2.0 to 256.0	29.8	70.9	168.2
SKBR-3	2.0 to 256.0	28.8	55.8	108.2

Effective doses (ED) values were calculated with Calcusyn software using the median effect principle defined by Chou and Talalay [26].

Although one cannot completely rule out the possibility that off-target ILK-independent, 267-mediated cellular effects might influence cell viability, treatment with 267 did cause dose dependent decreases in P-AKT levels, a key downstream target of ILK. These data have been summarized in Figure 1b, which provides the dose of 267 required to achieve 50% reduction of P-AKT in each of the seven cell lines evaluated. Cells were treated with eight different concentrations of 267 for eight hours and P-AKT levels in cell lysates were determined by western blot analysis as described in the Materials and methods. Dose-response curves were generated and the ED capable of eliciting a 50% decrease PAKT was extrapolated from individual curves. KPL4 cells did not exhibit any reductions in P-AKT even at the highest dose tested (50 μM). It is notable that suppression of P-AKT did not necessarily correlate with the cell viability data. For example, SKBR3 cells were quite sensitive to 267-mediated inhibition of P-AKT levels, but were the least sensitive in terms of the cell viability assessments as determined by Alamar Blue metabolic assay.

### Combination of 267 with chemotherapeutic agents commonly used for treating breast cancer identifies synergistic interactions with docetaxel

For an initial screen of drug combination effects two of the seven breast cancer cells (LCC6 and LCC6^Her2^) were treated with 267 in combination with cisplatin, doxorubicin, paclitaxel, vinorelbine, Dt, and Tz and cell viability was determined using the Alamar Blue metabolic assay. The combination effects were measured over a broad range of effective doses and the results have been summarized in Table 2. Importantly, combinations of 267 with Dt exhibited synergistic interactions at all drug ratios examined. In contrast, combinations of 267 with cisplatin, doxorubicin, paclitaxel, and vinorelbine exhibited antagonistic interactions. Tz exhibited variable interactions with 267, which appeared to be highly ratio dependent, a common feature associated with other drug combinations [35]. It should be noted, because Tz exhibited little measurable activity under the *in vitro *assay conditions used, fixed drug ratios of 267 with Tz were defined using the ED_50 _value of 267 and the maximum concentration of Tz that had been used in the single agent assay (1 mg/ml).

**Table 2:** Synergy, antagonism, and additivity in LCC6 and LCC6Her2 cells treated with QLT0267 in combination with several clinically relevant agents

**Chemotherapeutic agent**	**Drug concentration range**	**QLT0267: Drug ratio (nM)^1^**	**Result^2^**
Cisplatin	0.15 to 20.0 μM	1, 2, 4: 1	Antagonistic at all ratios
Doxorubicin	0.0625 to 2.0 μM	10, 20, 40: 1	Antagonistic at all ratios
Paclitaxel	0.313 to 15.0 nM	1000, 4000:1	Antagonistic at all ratios
Vinorelbine	0.0625 to 4.0 nM	10,000, 20,000, 40,000: 1	Antagonistic at all ratios
Docetaxel	0.125 to 1.0 nM	50,000, 95,000: 1	Synergistic at all ratios
Trastuzumab	3.1 × 10^-7 ^to 1 mg/ml	50,000: 1 mg/ml	Variable

^1 ^Drug ratios were chosen based on effective dose values of single agent treatment.

^2 ^Synergy is defined as a combination that exhibits combination index values of less then one at high affect levels over a minimum of four drug concentrations.

**Figure 2:**
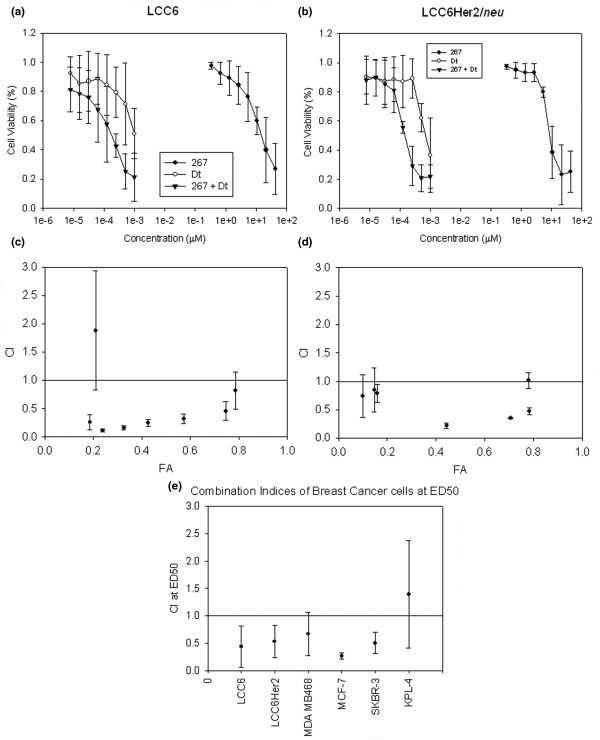
Breast cancer cells treated with 267 and docetaxel combined at a fixed ratio and added at various concentrations exhibit synergistic effects based on a measured cell viability endpoint. **(a) **LCC6 and **(b) **LCC6^Her2 ^cells were treated with increasing concentrations of 267, docetaxel (Dt) or 267 and Dt combined at a fixed ratio (50 μm:1 nm). Percentage cell viability relative to control cells not treated with drugs (100% viable) are shown and each data point is the average (± standard deviation) of triplicate samples. Combination index (CI) values determined by Calcusyn from dose-response curves of **(c) **LCC6 and **(d) **LCC6^Her2 ^cells treated with 267/Dt combinations. Data points represent the average (± standard deviation) from triplicate experiments. CI values less than one are indicative of synergistic effects; CI values greater than one are indicative of antagonistic effects; and CI values equal to one are indicative of additive effects. Fraction affected (FA) is a measure of the determined effect (cytotoxicity as measured by an Alamar blue assay) and amount of drug to achieve a FA of 0.5 is referred as the ED50. **(e) **Mean CI values at ED50 for LCC6, LCC6^Her2^, MCF-7, MDA MB468, KPL-4, and SKBR-3 cells treated with 267 and Dt combined are shown and each data point represents the average (± standard deviation) from triplicate experiments.

As shown in Figure 2, comparisons of dose response curves (cell viability) of LCC6 (Figure 2a) and LCC6^Her2 ^(Figure 2b) cells treated with 267 and Dt alone and in combination showed that when used in combination there was a shift in the dose-response curves to the left when the doses plotted for the combination are defined by the most active agent in the combination (Dt). Although statistically significant shifts in dose-response curves can be indicative of synergistic interactions, it is difficult to draw this conclusion on the basis of the sigmoidal dose response curves alone. Thus the dose-response data were analyzed using the MEP developed by Chou [26,33] (see Materials and methods). Using the CalcuSyn^® ^program, CI values were estimated and these results have been summarized in Figures 2c and 2d. The CI values for 267/Dt combinations were, in general, below 0.9 for both LCC6 (Figure 2c) and LCC6^Her2 ^(Figure 2d) treated cells, indicating weak to strong synergistic interactions. Importantly, the CI values were consistently below one over a broad range of effective doses as define by the fraction affected (FA) value.

The combination of 267 and Dt was also evaluated in several other breast cancer cell lines. CI values were calculated from cell viability dose response curves. These data are summarized in Figure 2e, which shows the CI values determined at the ED50 (dose exhibiting 50% loss in cell viability relative to untreated cells). The results indicate that the observed synergistic interactions (defined by mean CI values < 0.9) are achieved in at least five of the six cell lines tested (LCC6, LCC6^Her2^, MCF-7, MDA MB468, and SKBR-3). For KPL-4 cells the calculated CI values were indicative of slightly antagonistic (mean CI of 1.4) interactions.

**Figure 3:**
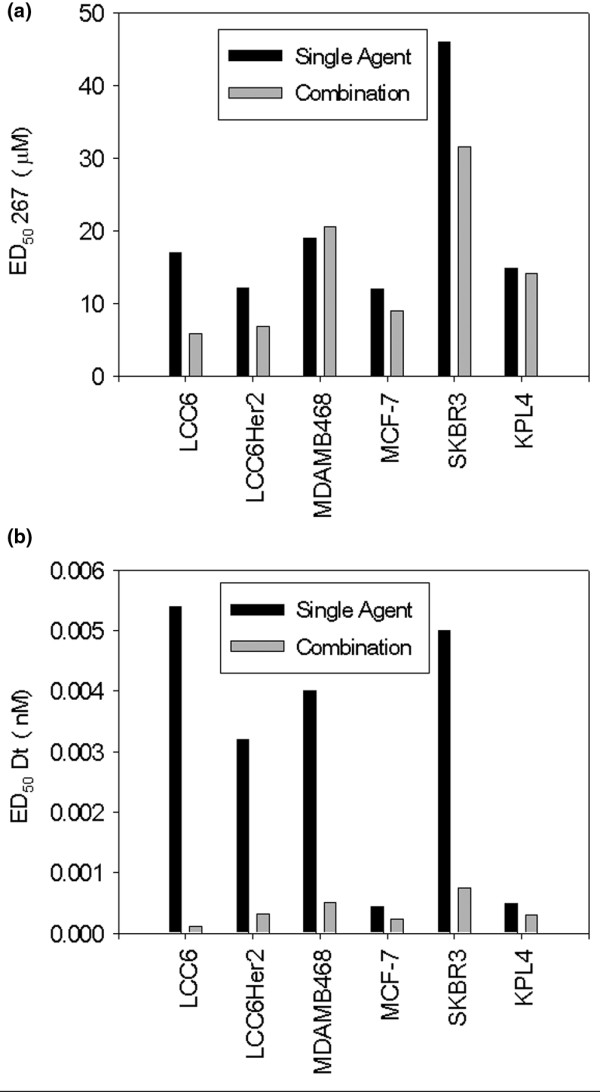
The dose reduction index (DRI) calculated using the Calcusyn program was used to estimate the ED50 of drugs (267 and/or docetaxel) against the indicated cell lines. The DRI estimates the extent to which the dose of one or more agents in the combination can be reduced to achieve effect levels that are comparable with those achieved with single agents. Black bars indicate the mean ED50 calculated for the drugs when added alone and the grey bars indicate the mean ED50 calculated for the drugs when used in combination. **(a) **ED50s for QLT0267 (267); when used in combination to treat LCC6 cells the ED50 of 267 can be reduced by 3.6-fold. **(b) **ED50s for docetaxel; the dose reduction index assessment for docetaxel (Dt) indicated that dose reductions of up to 27.5 fold (LCC6 cells) can be obtained.

If drug combinations interact in a manner that result in synergy, then the dose of each drug used in the combination to achieve a specific measurable effect level will be substantially reduced when compared with the dose needed to achieve the same effect level when the drugs are given alone. This parameter can be calculated and is defined by the DRI (refer to Materials and methods). The DRI can be used to estimate the doses of 267 and Dt needed when used in combination to achieve a defined effect level which can then be compared with the single agent dose required to achieve this effect. Based on these analyses, it was estimated that the concentration (dose) of 267 in the 267/Dt combination required to achieve an ED50 could be reduced by up to 3.6-fold in the LCC6 cell line (Figure 3a). 267 dose reductions were less remarkable in the other cell lines evaluated; ranging from no change to a 30% reduction. A similar analysis was completed for Dt (Figure 3b) and it was estimated that the concentration (dose) of Dt in the 267/Dt combination required to achieve an ED50 could be reduced in all cell lines by 2 to 25-fold when compared with Dt alone. For example in SKBR3 cells the ED50 of Dt given alone is 5 nM while in combination with 267 the ED50 of Dt decreases to less than 1 nM.

### 267 and 267/Dt combination treatments cause dose-dependent reduction in P-AKT levels estimated by western blot analysis

**Figure 4:**
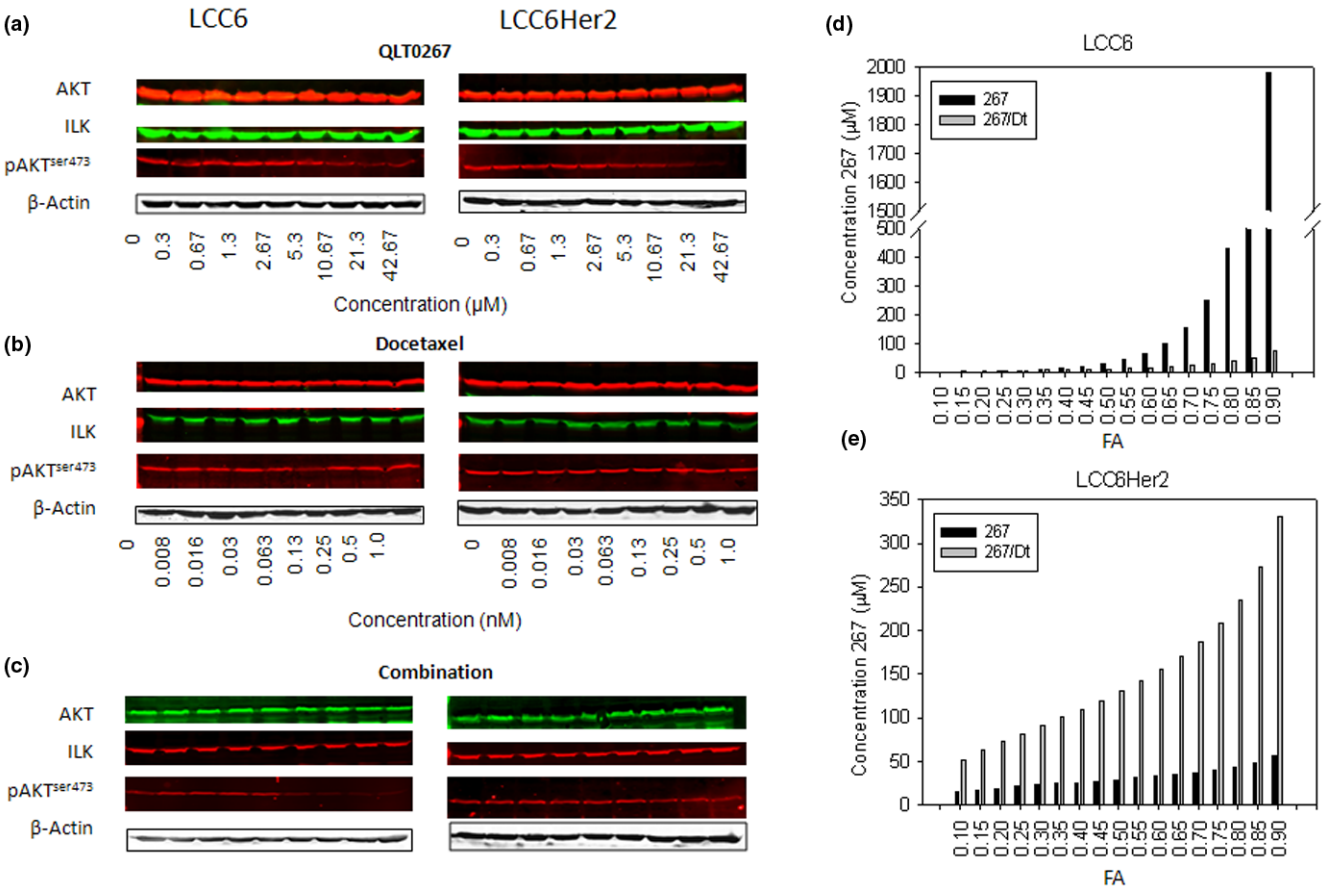
Dose response curves were generated for changes in P-AKT in LCC6 and LCC6^Her2 ^cells after treatment with QLT0267, docetaxel, or a fixed ratio combination of QLT0267 and docetaxel. LCC6 and LCC6^Her2 ^cells were treated for eight hours with increasing concentrations of QLT0267, docetaxel, or a fixed ratio combination of QLT0267 and docetaxel (50 μm:1 nm) to establish dose response curves based on an endpoint measuring suppression of P-AKT levels as determined by western blot analysis. Representative western blot images were collected using a fluorescence based imaging system (odyssey, Licor) where the colours (red or green) represent the secondary antibody used, show that increasing concentrations of **(b) **docetaxel (Dt) exerted no significant effect on the expression of integrin-linked kinase (ILK), protein kinase B (AKT), and phosphorylated protein kinase B (P-AKT) in either cell line. Treatment with increasing doses of **(a) **QLT0267 (267) alone or **(c) **in combination with Dt showed dose-dependent decrease in P-AKT. Densitometry assessment of western blots (n = 3) were used to estimate treatment response relative to controls (taken to be 100% P-AKT levels) and the resulting data was then analyzed by Calcusyn to determine estimated DRI. The DRI was then used to estimate the dose of 267 when used alone (black bars) or in combination with Dt (grey bars) needed to achieve a defined fraction affected (FA). The dose of drug required to achieve an FA of 0.5 is defined as the ED50 for the measured P-AKT suppression endpoint. The ED50 of 267 was about 30 μM in the **(d) **LCC6 cell line, while in the presence of **(e) **Dt the 267 ED50 was about 11 μM. In LCC6^Her2 ^cells, more 267 was required when used in combination to achieve FA similar to that of single agents. For example in the LCC6^Her2 ^cells, the 267 ED50 when used alone was about 30 μM, and this increased to 130 μM when 267 was used in combination with Dt.

Western blot analysis was used to assess P-AKT (specifically at serine 473) levels in LCC6 and LCC6^Her2 ^cells treated with increasing concentrations of 267 alone (Figure 4a), Dt alone (Figure 4b), or 267 in combination with Dt (Figure 4c). In these studies P-AKT (Ser473) was measured eight hours after addition of 267, a time point selected because no significant changes in cell viability (as measured by dye exclusion) were noted (even at the highest 267 dose used) yet significant reductions in P-AKT (Ser473) were detectable as noted in the representative western blots shown in Figure 4. P-AKT (Ser473) levels were reduced in a dose-dependent manner over the range of 267 concentrations evaluated in both LCC6 and LCC6^Her2 ^cells (Figure 4a). Dt treatment alone was shown to have little or no measurable effect on P-AKT (Ser473) levels (Figure 4b). In cells treated with the 267/Dt there were significant reductions in P-AKT (Ser473) levels which were also dose dependent (Figure 4c). None of the treatment strategies were shown to influence expression of total ILK or total AKT where protein loading was verified using β-actin. P-AKT levels from three independent experiments (as illustrated in Figures 4a to 4c) were qualitatively assessed by densitometry to estimate the effective doses needed to achieve a defined effect level represented by a FA value. As described above, these data in turn, could be used to estimate the dose of 267 required to achieve a defined level of P-AKT (Ser473) suppression when the drug was used alone or in combination with Dt. These calculated values have been summarized in Figure 4d (LCC6) and 4e LCC6^Her2^). The results clearly demonstrate that the combination acts differently in the Her2-positive cell line when compared with the parental LCC6 cell line. More specifically for LCC6 cells the dose of 267 required to achieve a defined level of P-AKT (Ser473) suppression was substantially reduced when Dt was present indicating that Dt potentiates 267 mediated suppression of P-AKT (Ser473). For example, the dose of 267 required to achieve 50% suppression of P-AKT (Ser473) (FA = 0.5) when used alone was calculated to be 30 μM, while in combination with Dt the dose required to achieve the same FA was reduced three-fold. In contrast, the densitometry data indicated that for LCC6^Her2 ^cells, the concentration of 267 required in combination with Dt to achieve a defined effect (FA) on P-AKT (Ser473) inhibition was significantly higher than that required when 267 was used as a single agent. For example, 30 μM 267 was required to achieve an FA of 0.5 (ED50) when 267 was used alone; however, in the presence of Dt the concentration of 267 required to achieve an FA of 0.5 was estimated to be 130 μM.

**Figure 5:**
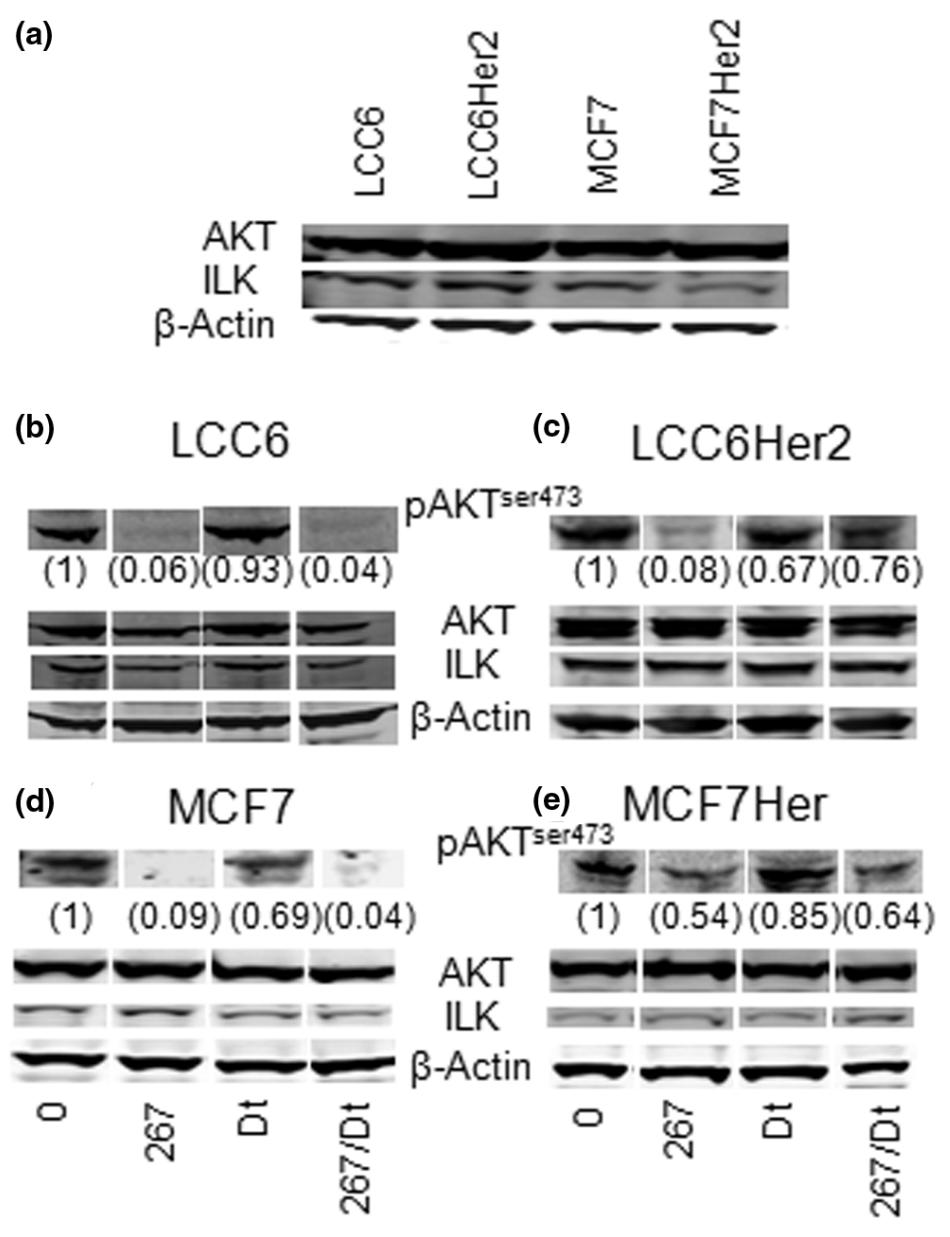
Levels of P-AKT were measured in LCC6, LCC6^Her2^, MCF-7, and MCF-7^Her2 ^cells after treatment with a singles dose of QLT0267, docetaxel, or a combination of QLT0267 and docetaxel. **(a) **LCC6, LCC6^Her2^, MCF-7, and MCF-7^Her2 ^cells express baseline levels of integrin-linked kinase (ILK), protein kinase B (AKT), and phosphorylated AKT (P-AKT). **(b) **LCC6, **(c) **LCC6^Her2^, **(d) **MCF-7, and **(e) **MCF-7^Her2 ^cells were treated for eight hours QLT0267 (267) (42 μM), docetaxel (Dt) (1 nM), or the combination of 267 and Dt. Western blot analysis using a fluorescence based imaging system (odyssey, Licor) shows that treatment with 267 and 267/Dt elicit considerable reductions in the level of P-AKT relative to controls (untreated cells). Treatment with Dt had no effect on P-AKT levels. Reductions in P-AKT were not attributable to changes in the level of ILK or AKT. Band intensities for P-AKT were normalized to actin then to untreated controls and changes in levels are indicated in the values provided just below the P-AKT band (n = 3).

Differences in the combination effects due to Her2 over-expression were confirmed using the MCF-7 and MCF-7^Her2 ^cell lines, as summarized in the representative western blots shown in Figure 5. Qualitative (densitometry) assessments of the P-AKT (Ser473) western blot data (average of triplicate experiments) have been presented as a value that is relative to control (untreated) P-AKT levels (Ser473) and these are provided in brackets. The 267/Dt combination resulted in enhanced P-AKT (Ser473) suppression compared with 267 alone when used to treat the parental cell lines (Figures 5b, d). However, this combination effect was lost when tested in the Her2 over-expressing cell lines, where the level of P-AKT (Ser473) suppression was no better or even worse than when 267 was used alone (compare Figures 5c and 5e). This effect is most notable in the LCC6^Her2 ^cells where 267 caused a 92% reduction in P-AKT (relative to control) when used alone, but only a 24% reduction when used in combination with Dt. It should be noted that all four cell lines studies expressed similar levels of ILK and AKT (Figure 5a) and treatment with 267 and Dt alone or in combination did not effect total ILK or AKT levels as detected by western blot analysis.

### 267 and 267/Dt combinations inhibit VEGF secretion

**Figure 6:**
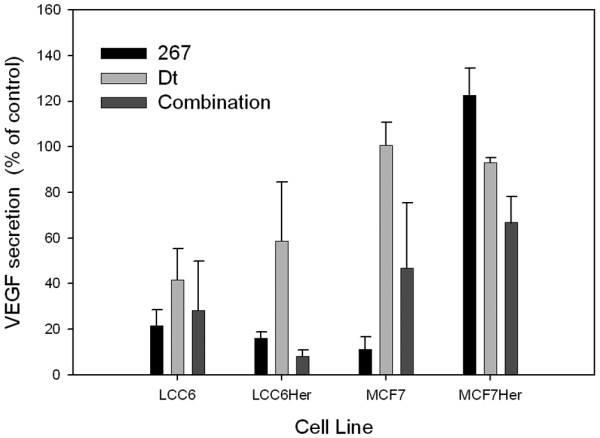
Levels of secreted VEGF were measured in LCC6, LCC6^Her2^, MCF-7, and MCF-7^Her2 ^cells after treatment with a singles dose of QLT0267, docetaxel, or a combination of QLT0267 and docetaxel. LCC6, LCC6^Her2^, MCF-7, and MCF-7^Her2 ^were treated with 267 (10 μM), Dt (0.25 nM) or a combination of both for 72 hours. Conditioned media was collected subsequently assessed for vascular endothelial growth factor (VEGF) secretions using ELISA as described in the Materials and methods. All four cell lines assessed showed a decrease in VEGF secretion when treated with low doses of QLT0267 (267) alone or with the combination 267/docetaxel (Dt). Dt also moderately attenuated VEGF secretion in each cell line (n = 3).

We investigated whether 267 alone or in combination with Dt could influence VEGF secretion in LCC6, LCC6^Her2^, MCF-7, and MCF-7^Her2 ^cells, an endpoint measured 72 hours after drug addition. The 72-hour time point was selected because VEGF levels in the media were highest at this time; however, it can be suggested at this time point VEGF levels would be a reflection of both direct effects of 267 on VEGF expression and indirect effects due to 267 and/or Dt cytotoxicity as fewer viable cells capable of producing VEGF would be present. For this reason we focused on doses of 267 and Dt below that which caused 50% toxicity over the 72-hour incubation time. The results, summarized in Figure 6, are consistent with previous publications and indicate that when LCC6, LCC6^Her2^, and MCF-7 cells are treated with 267 there is a significant decrease in VEGF secretion. This decrease was not observed in the MCF-7^Her2 ^cell line. Treatment of LCC6 and LCC6^Her2 ^cells with 10 μM 267 resulted in an approximately 79% and 83% decrease in VEGF secretion, respectively. When Dt was combined with 267, the decrease in VEGF secretion was larger (92%) when the drugs were added in combination to the LCC6^Her2 ^cells. Conversely, when the drugs were used in combination to treat the LCC6 cells the decrease in VEGF levels in the media was 72%, an effect that was actually less then what was observed when using 267 alone. It should be noted that treatment with Dt (0.25 nM) was associated with a 56% and a 40% decrease in VEGF levels relative to controls for the LCC6 and LCC6^Her2 ^cells, respectively. Thus the enhanced effect observed when using 267/Dt combination against the LCC6^Her2 ^cells could be explained by the effects of the individual agents. This, however, is not the case for the LCC6 cells. The effect of 267 on VEGF secreted by MCF-7 cells was similar to that observed with the LCC6 cell line; 267 produced a 90% reduction in VEGF secretion when used alone and only 53% reduction when used in combination with Dt. Results obtained with the MCF-7^Her2 ^cell line suggest that significantly higher doses of 267 (more than 21 μM) was required to see changes in VEGF levels found in the media. However, when MCF-7^Her2 ^cells are treated with a combination of 267 (10 μM) and Dt (0.25 nM) significant reductions in VEGF secretion were seen.

### 267/Dt treatment causes disruption of normal F-actin cytoarchitecture and abnormal nuclear morphology

**Figure 7:**
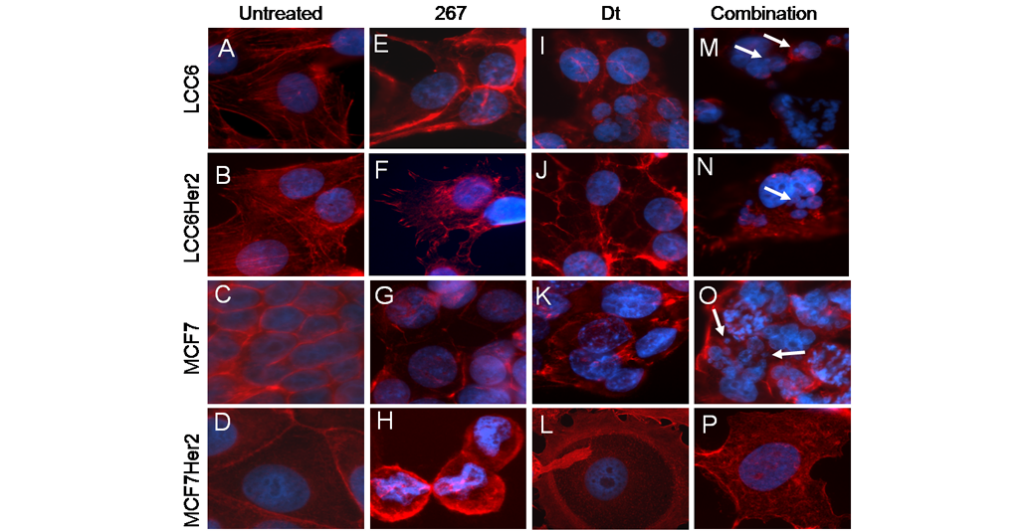
267/Dt treatment of LCC6, LCC6^Her2^, MCF-7, and MCF-7^Her2 ^causes disruption of normal F-actin cytoarchitecture and abnormal nuclear morphology. LCC6, LCC6^Her2^, MCF-7, and MCF-7^Her2 ^were treated for eight hours with 267 (42 μM), Dt (1 nM) or the combination of 267 and Dt. Subsequently, cells were fixed using paraformaldehyde, permeabilized, and stained for nuclear material using Hoechst and F-actin using Texas red conjugated phalloidin. Representative photomicrographs of **(a to d) **untreated cells or cells treated with **(e to h) **QLT0267 (267), **(i to l) **docetaxel (Dt) and **(m to p) **the combination of 267/Dt are shown, where blue represents the nucleus and red the F-actin microfilaments. Combination treatment resulted in a distinct decrease in total F-actin staining, a change in actin organization, the appearance of apoptotic nuclear bodies (white arrows), as well as metaphase chromosomes suggestive of a cell cycle block in these cells.

In addition to assessing how Dt influenced known or suspected downstream effects of the action of 267 on ILK (P-AKT levels, VEGF secretion), the influence of 267 on Dt-induced changes in cytoarchitecture and nuclear morphology were investigated eight hours after drug addition to the cells. The drug doses used were 42 μM for 267 and 1 μM for Dt; dose that are cytotoxic (Alamar Blue metabolic assay) after 72 hours but exhibit no significant cytotoxicity at eight hours after drug addition. As illustrated by the representative photomicrographs in Figure 7, immunofluorescence based experimentation showed that untreated LCC6 (Figure 7a) and LCC6^Her2 ^(Figure 7b) cells contained normal intact nuclei (blue color) and typical F-actin cytoskeleton (red color) with distinct intracellular organization and prominent stress fibers. LCC6 cells treated with 267 alone (Figure 7e) showed an accumulation of F-actin at the cell periphery, while LCC6^Her2 ^cells treated with 267 alone (Figure 7f) exhibited cytoplasmic actin distribution and increased formation of focal adhesions at cell periphery. As expected, Dt treatment alone in LCC6 and LCC6^Her2 ^cells caused significant degeneration of both F-actin microfilaments (Figures 7i and 7j). Importantly, 267/Dt treated LCC6 and LCC6^Her2 ^cells (Figures 7k and 7l, respectively) showed more pronounced reduction of F-actin, appearance of apoptotic nuclear bodies (arrows), and metaphase chromosomes, suggesting that 267/Dt combination in these cell types specifically inhibited cell cycle progression.

Untreated MCF-7 cells showed the typical cytoplasmic distribution of F-actin slightly enriched at the cellular membrane and lack stress fibers (Figure 7c). MCF-7 cells treated with 267 (Figure 7g) showed accumulation of F-actin at the cell periphery and punctate cytoplasmic staining, although cells treated with Dt alone (Figure 7k) showed decreased F-actin expression, loss of uniform expression, and increased punctate areas. Images of MCF-7 cells treated with 267/Dt (Figure 7o) were strikingly similar to those shown for LCC6 cells treated with this combination; reflected by reduced F-actin distribution, appearance of apoptotic nuclear bodies (arrows), and presence of metaphase chromosomes. Untreated MCF-7^Her2 ^cells showed typical punctate and peripheral staining of F-actin as well as large nuclei enriched localization of F-actin at the cell membrane (Figure 7d). MCF-7^Her2 ^cells treated with 267 alone showed cell rounding and enriched F-Actin at the cell membrane (Figure 7h), while cells treated with Dt alone showed trademark [36] F-actin rings, peripheral stress fibers, and punctate cytoplasmic staining (Figure 7l). Finally MCF-7^Her2 ^cells treated with 267/Dt (Figure 7p) showed disorganized F-actin, with peripheral staining; however, in contrast to all the other cell lines examined, only minor changes in nuclear morphology were evident.

### 267/Dt combination therapy *in vivo *correlates with reduced tumor burden and extended survival in orthotopic LCC6 breast cancer tumor model

**Figure 8:**
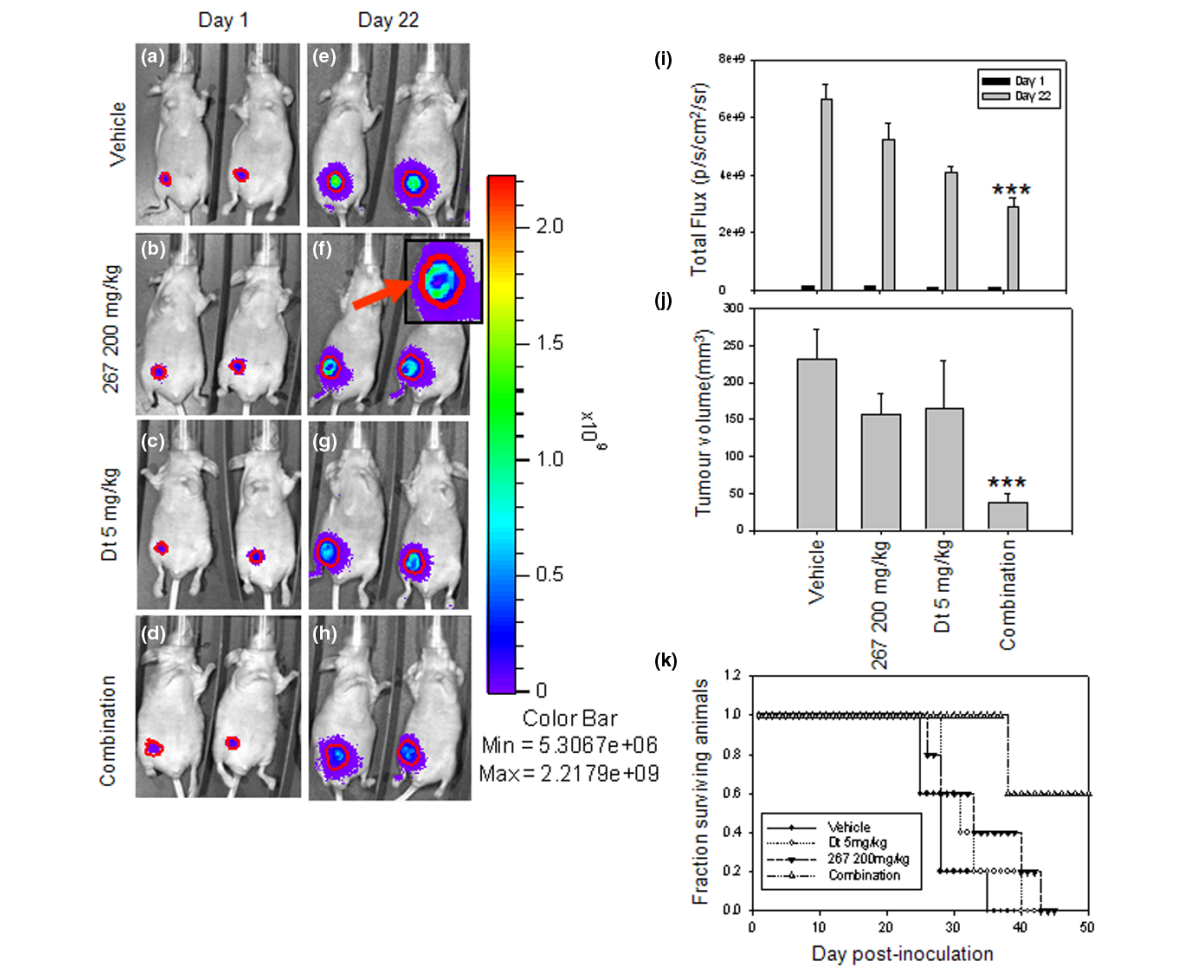
267/Dt combination therapy *in vivo *correlates with reduced tumor burden and extended survival in orthotopic LCC6 breast cancer tumor model. Bioluminescent imaging of orthotopic LCC6 tumors are shown one day after treatment initiation and on day 22 after treatment initiation. The treatment groups included **(a and e) **vehicle controls, **(b and f) **QLT0267 (267), **(c and g) **docetaxel (Dt), and the **(d and h) **combination of 267/Dt treated animals (doses indicated on graph). Total light emission from tumors in animals was visualized and quantified. **(i) **Animals treated with the combination showed lower total light flux than all other treatment groups. Animals treated with 267 exhibited tumors with darkened areas in the core (see inset in f as an example). Tumor size in treated animals was measured by callipers and these data were used to estimate the **(j) **tumor volumes. The combination of 267/Dt was significantly lower (***P < 0.005) then all other treatment groups analyzed. **(k) **Kaplan-Meier survival analysis of data defining survival endpoints based on tumor ulceration and/or tumors more than 0.5 g were used to determine median survival times. For animals treated with 267 (200 mg/kg) the median survival time was 33 days (26 days post-treatment initiation), animals treated with Dt (5 mg/kg) exhibited a median survival time of 31 days (24 days post-treatment initiation); and animals treated with the 267/Dt combination exhibited a median survival time of more than 90 days (83 days post-treatment initiation). In this group three out of five animals were alive at day 90, while no animals were alive for any other treatment group.

The results presented thus far indicate that combinations of 267 and Dt should provide improved therapeutic effects based on several different therapeutically relevant endpoints when used to treat breast cancers with low Her2 expression. The results demonstrated that the combination effects are more complicated in cell lines that over-express Her2 and that for some endpoints measured (P-AKT) the data do not necessarily support further development of the 267/Dt combination for tumors that over-express Her2. Studies to be reported elsewhere have been completed to better characterize the effects of 267 and ILK inhibition in Her2 over-expressing cell lines. Here, however, we determined whether the favourable drug-drug interactions observed *in vitro *for the low Her2 expressing cells line could be recapitulated *in vivo*. 267 and Dt alone and in combination were used to treat mice with established LCC6^luc ^tumors (LCC6 transfected with luciferase as described in the Materials and methods). These tumors were readily detectable in all mice 24 hours and seven days post-implantation of 2 × 10^6 ^cells. Mice (n = 5, per treatment strategy) were treated with: the vehicle controls used for both 267 (orally) and Dt (intravenously); 200 mg/kg 267 (QD once daily for 28 days; orally); 10 mg/kg Dt (Q7D once weekly for four weeks; intravenously); or 267 (200 mg/kg; orally)/Dt (5 mg/kg; intravenously). The 267 dose and schedule was selected based on previous studies that showed effective therapy in different human xenograft models [21,37]. The aim of this study was to determine whether use of 267 (200 mg/kg QD once daily for twenty eight days) in combination with Dt might improve treatment outcomes. A suboptimal dose of Dt (5 mg/kg) was administered using a Q7D once a week for four weeks dose schedule in order for us to assess whether 267 contributed to improved outcomes in a combination setting. The results of this *in vivo *efficacy study have been summarized in Figure 8. Tumor growth was monitored using non-invasive imaging (Figures 8a to 8i) using the IVIS 200 (Xenogen, Caliper Life Sciences, MA, USA) to image luciferase expressing LCC6 cells and by external calliper measurements (Figure 8j). Survival (Figure 8k) was determined based on the time in days required for the mice to be terminated due to tumor ulceration and/or the presence of tumors exhibiting volumes in excess of 500 mg.

Tumors in animals treated with 267 (Figure 8f), Dt (Figure 8g), and 267/Dt (Figure 8i) all showed reduced total light emission (luciferase expression) 22 days post-cell injection when compared with vehicle-treated control mice (Figure 8e). Quantification of total light flux (n = 5 mice per treatment, average ± standard error, background subtracted; Figure 8i) demonstrated tumor burden was significantly less in mice that had received the combination treatment as compared with mice treated with the vehicle control or 267 alone (*P *< 0.05). There was a modest difference in tumor burden between Dt and 267/Dt-treated mice, but this difference was not statistically significant. When tumor burden was measured using callipers, the tumors from 267/Dt-treated mice were significantly smaller compared with all other treatment groups, including mice treated with Dt alone, (*P *< 0.05; Figure 8j). It is interesting to note that close examination of the pattern of luciferase expression showed that tumors from 267-treated animals (alone or in combination) exhibited dark regions in the center of the tumor (for an example refer to Figure 8f, inset). These dark regions may reflect regions of necrosis or alternatively could be a result of treatment induced changes in tumor perfusion that may alter luciferin delivery to the tumors.

Kaplan-Meir survival analysis (Figure 8k) based on survival endpoints defined by tumor ulceration and/or tumor size (500 mg or larger) showed that the median survival time was 28 days (or 21 day post-treatment initiation) for untreated mice, 33 days (or 26 days post-treatment initiation) for mice treated with 267, 31 days (or 24 days post-treatment initiation) for mice treated with Dt and more than 90 days (or > 83 days post-treatment initiation) for mice treated with the 267/Dt combination. In reference to the latter group, it should be note that three out of five mice treated with 267/Dt combinations were still alive at day 91, while mice from all other treatment groups had been terminated due to tumor ulceration and/or a tumor size of more than 500 mg.

## Discussion

Although it is understood that ILK is an important therapeutic target in cancer, the data summarized here (Figure 8) and elsewhere suggest that an ILK inhibitor such as 267 given alone will not achieve much more than a delay in tumor progression. Lack of potent single-agent activity, when using *in vivo *tumor growth as an efficacy measure, lends support to the belief that ILK inhibitors must be developed in the context of other therapeutics. A similar trend was exemplified by treatment regiments incorporating Tz (Herceptin™), a therapy that targets Her2-expressing tumors. Tz as a single agent exhibits little significant activity, but when used in a combination setting it has proved to be of significant therapeutic value [38]. The studies described here, focused on identifying agents that would work synergistically with QLT0267. We used cell-based screening assays in order to assess whether drugs commonly used for breast cancer could be combined with 267 to achieve better then expected therapeutic results. For these studies a fixed-drug ratio experimental design was used where drug-drug interactions were determined using at least three different drug-drug ratios applied over a broad range of effective doses (Table 2). We show for the first time that combination of 267/Dt appeared to interact in a manner that results in synergy. Drug-drug interactions were measured by use of the median effect method of Chou and Talalay [26] and were initially determined on the basis of a therapeutic endpoint measuring metabolic activity (Alamar Blue assay). Synergy was observed over a broad range of effective dose and was measured in five out of six breast cancer cell lines tested (Figure 2), regardless of Her2 status. Although limited to results obtained with the two cell lines used for the broad combination screen (LCC6 and LCC6^Her2^) it is interesting to note that the 267/Dt combination was synergistic while combinations of 267 with paclitaxel and vinorelbine appeared antagonistic. This would suggest that the mechanism(s) promoting synergy may not involve microtubules in general. It has been suggested that Dt is more effective in treatment of breast cancer than paclitaxel [39] and in addition to its influence on microtubule assembly that culminates in a general cytotoxic response, Dt activity has been linked to increased activation of the apoptotic program and to changes of apoptotic marker expression [40-43]. It may be these additional activities of Dt that combine with 267 to produce enhanced therapeutic effects.

It was important to demonstrate that the individual drugs within the 267/Dt combination exert benefits consistent with their individual mechanisms of action. For example, 267 activity can be linked to measured changes in P-AKT (ser473) levels and VEGF while Dt activity can be assessed by drug-mediated changes in cell architecture. ILK inhibition by 267 engenders dose dependent decreases in levels of P-AKT (Figures 1b, 4 and 5) and when 267 is added as a single agent it can inhibit VEGF secretion (Figure 6). Perhaps unexpectedly, single-agent 267 treatment also caused changes in cytoarchitecture and nuclear morphometry (Figure 7). This effect of 267 has not be reported previously, however, studies have provided evidence that ILK plays a role in cytoskeletal arrangement of actin through the regulation of proteins such as Rac and Cdc42 [9,44,45]. Furthermore, siRNA mediated ILK silencing resulted in diminished cell spreading and actin cytoskeleton reorganization; results that help to explain ILK's role in the regulation of cancer cell motility and invasiveness [46]. Recent evidence indicates a role for ILK in regulation of mitotic spindle organization [47]. When this information is considered in light of the activity of Dt, one can speculate about the mechanism that may be promoting synergy when Dt is used in combination with 267. Studies have shown that cells treated with Dt exhibit a reorganization of the microfilament network [36], disturbed microtubule structures, less F-actin stress fiber formation, decreased activation of Rac1/Cdc42, reduced cell motility, and an inhibition of angiogenesis [48]. When considering the primary effect of Dt on the microtubule cytoskeleton of cancer cells, and based on the results summarized here it can be suggested that the combination of Dt and 267 may result in synergistic changes in tubulin, F-actin organization, and nuclear degeneration during apoptosis.

As indicated above, inhibition of ILK by 267 was expected to cause a decrease in P-AKT at serine 473. However, the effect of Dt on AKT has not been well studied. Studies have suggested that Dt can suppress the phosphorylation of AKT in lymphoma cell lines [49] and lung carcinoma [50]. Others have suggested that the AKT pathway can be activated by Dt [51]. As shown in Figure 4, results obtained in several breast cancer cell lines indicate that Dt added at doses of up to 1 nM exerted no significant effect on P-AKT levels after an eight-hour exposure. Importantly, Dt potentiates the effect of 267 on P-AKT levels, at least in LCC6 and MCF-7 cell lines (Figures 4 and 5). Interestingly, this beneficial combination effect was not observed in the Her2 transfected variants of these cell lines, suggesting that phosphorylation of AKT does not play a role in the enhanced cytototoxicity seen when 267 is combined with Dt to treat the Her2 over-expressing cells.

It has also been established that one of the beneficial therapeutic effects of 267 is associated with its ability to inhibit VEGF secretion. More specifically, it has been reported that integrins cooperate with the VEGF receptors to promote angiogenesis in vascular endothelial cells [52] and other studies indicate that ILK and PI3-kinase are involved in VEGF signaling pathways [53]. Although not well studied, it has been suggested that Dt can influence vascularization *in vivo *in a fashion that is related to VEGF signaling. More specifically, Murtagh and Schwartz [54] have recently demonstrated that Dt can prevent VEGF-induced phosphorylation of focal adhesion kinase, Akt and endothelial nitric oxide synthase, effects that may be mediated by Dt mediated dissociation of Hsp90 from tubulin and subsequent Hsp90 degradation by ubiquination. Thus, it could be speculated that combinations of 267 and Dt would be of particular interest in the context of VEGF-induced tumor vascularization; where 267 would suppress VEGF production and Dt would mitigate signaling through any remaining VEGF. However, preliminary *in vitro *studies summarized in Figure 6 suggest in the cell lines that express low levels of Her2 that the 267/Dt combination was less effective at inhibiting VEGF secretion then when 267 was used alone. Similar to the P-AKT (Ser473) results, when using VEGF secretion as an endpoint, the results obtained in the Her2 over-expressing cell lines differed from those obtained with cells that express low Her2 levels.

On the basis of VEGF secretion and P-AKT (Ser473) data we can conclude that the 267/Dt drug combination effects were dependent on Her2 expression. These differences encouraged us to assess the effect of 267 on Her2 signalling in the Her2-positive cell lines. Although not reported here, these studies demonstrated that 267 treatment induced a dose-dependent decrease in Her2 levels; an effect that could also be obtained when using siRNA to silence ILK. This unexpected effect of 267 on Her2-positive cell lines complicated the interpretation of results in these cells and for this reason the *in vivo *studies reported here focused on mice bearing orthotopically transplanted LCC6 cells, which do not express detectable levels of Her2.

## Conclusions

This *in vivo *study provided evidence supportive of the beneficial therapeutic effects of the 267/Dt combination LCC6 tumors (Figure 8) and suggest that further studies are warranted to address development of this combinations and the factors that may influence treatment outcomes; factors that include drug dose, schedule and sequencing as well as an assessment of therapeutic response *in vivo *that also includes multiple endpoints (e.g. tumor cell proliferation and death as well as tumor vascularization).

## Abbreviations

267: QLT0267; AKT: protein kinase B; BSA: bovine serum albumin; CH-ILKBP: calponin homology-containing ILK-binding protein; CI: combination index; D: dose; DMEM: Dulbecco's modified eagle's medium; DRI: dose reduction index; Dt: docetaxel; ECM: extracellular matrix; ED: effective doses; EDTA: ethylenediaminetetraacetic acid; EMT: epithelial to mesenchymal transition; F-actin: filamentous actin; FA/fa: fraction affected; FBS: fetal bovine serum; fu: fraction unaffected; GFP: green fluorescent protein; GSK-3: glycogen synthase kinase 3; Her2: human epidermal growth factor receptor 2; ILK: integrin-linked kinase; LCC6: MDA MB435/LCC6; LCC6Her2: MDA MB435/LCC6^Her2/*neu*^; luc: luciferin; MEP: median effect method; MLC: myosin light chain; P-AKT: phosphorylated AKT (serine 473); PBS: phosphate buffered saline; PDK-1: phosphoinositide-dependent kinase 1; ROI: region of interest; Tz: trastuzumab; v/v: volume to volume; VEGF: vascular endothelial growth factor; w/v: weight to volume.

## Competing interests

The authors declare that they have no competing interests.

## Authors' contributions

JK designed and executed all experiments and data analysis and wrote the manuscript. CW contributed the LCC6^Her2 ^cell line, executed confirmatory studies, and helped revise the manuscript. KF conducted initial exploratory studies to identify drug combinations. LE contributed to study design. TD contributed QLT0267. DW, and BS helped write the manuscript. KG gave clinical expertise. WD and SD helped revise the manuscript. MB is Chief Investigator, conceived the study design and helped write the manuscript.
